# Supplementary material for: Fresh groundwater discharge insignificant for the world’s oceans but important for coastal ecosystems
Source: Nat Commun. 2020 Mar 9;11:1260. doi: 10.1038/s41467-020-15064-8 (PMC7062736; doi:10.1038/s41467-020-15064-8)
Supplement: Supplementary file 1 — Supplementary Information [file 41467_2020_15064_MOESM1_ESM.pdf]

Supplementary information for

Fresh groundwater discharge insignificant for the world's oceans but important for coastal ecosystems

By Luijendijk et al.

Supplementary Notes

Supplementary Note 1

We modeled coupled density-dependent groundwater flow and solute transport in coastal aquifers using a new model code, GroMPy-couple, which is a Python shell around the generic finite element code escript[1,2]. Escript is used to solve the governing equations for coupled solute transport and density-driven groundwater flow. The choice for escript was motivated by the fact that the code runs on parallel computing architecture, which strongly increases computational efficiency, and by the Python interface of escript, which provides a tool for the automated execution and analysis of large numbers of model runs. An alternative like the more well-known model code SEAWAT[3] was not used because the finite difference scheme used by SEAWAT does not allow local grid-refinement around an inclined fresh-salt water interface in coastal aquifers. While we did initially use the finite element code SUTRA[4], SUTRA at present does not offer parallel computation, has no Python interface to allow executing multiple model runs, and does not have facilities to halt transient model runs when they have reached steady-state. Escript has been used to simulate density-driven fluid flow and reactive transport in a number of studies[1,2,5,6], although this is to our knowledge the first time it has been applied to model coastal groundwater flow.

Density dependent groundwater flow was modeled by solving the following equation[7]:

$$S\frac{\partial P}{\partial t} + \phi\gamma\frac{\partial C}{\partial t} = \nabla\frac{\rho_f k}{\mu}\left(\nabla P + \rho_f g \nabla z\right) + Q_f \qquad (1)$$

Where S is storativity ($s^2 m^{-2}$), P is pressure (Pa), t is time (sec), C is solute concentration ($kg\ kg^{-1}$), ρ_f is fluid density ($kg\ m^{-3}$), ϕ is porosity (dimensionless), γ is solute expansion coefficient (dimensionless), k is permeability tensor (m^2), μ is dynamic viscosity *(Pa s)*, g is the gravitational constant (9.81 $m\ s^{-2}$), z is elevation (m) and Q_f is a fluid source or sink ($kg\ m^{-3}\ s^{-1}$). We used a value of storativity (S) of 1.0×10-4 Pa^{-1}. Solute transport is given by [7]:

$$\frac{\partial(\rho_f C)}{\partial t} = \nabla\rho_f D_h \nabla C - \mathbf{v}\nabla\left(\rho_f C\right) + \frac{Q_f C_s}{\phi} \qquad (2)$$

Where D_h is the dispersion coefficient tensor ($m^2\ s^{-1}$), **v** is flow velocity ($m\ s^{-1}$) and C_s is solute concentration of the fluid source or sink ($kg\ kg^{-1}$).The hydrodynamic dispersion tensor D_h is calculated as[8]:

$$D_{h,xx} = \frac{v_x^2}{|v|}\alpha_L + \frac{v_z^2}{|v|}\alpha_T + D_m \qquad (3a)$$

$$D_{h,zz} = \frac{v_x^2}{|v|}\alpha_T + \frac{v_z^2}{|v|}\alpha_L + D_m \qquad (3b)$$

$$D_{h,xz} = D_{h,zx} = \left(\alpha_L - \alpha_T\frac{v_x v_z}{|v|}\right) \qquad (3c)$$

Where α_L and α_T are the longitudinal and transverse dispersivity coefficient, respectively (m), and $|v|$ is the absolute value of groundwater velocity ($m\ s^{-1}$).

Flow velocity $\boldsymbol{v}$ is calculated as:

$$\boldsymbol{v} = \frac{k}{\mu\phi}\left(\nabla P + \rho_f g \nabla z\right) \qquad (4)$$

Fluid density and viscosity are calculated using a linear equation of state and assuming a constant temperature and pressure:

$$\rho_f = \rho_w + \rho_w \gamma C \qquad (5)$$

$$\mu = \mu_0 + aC \qquad (6)$$

Where ρ_w and μ_0 are the density (kg m^{-3}) and viscosity (Pa s) of pure water, respectively, and γ and a are dimensionless constants. We used values for ρ_w, γ, μ_0 and a of 998.872 kg m^{-3}, 0.6841, 9.808×10^{-4} Pa s, and 2.6515×10^{-3}, respectively. These values were found using linear regression to published equations of state[9] over the range of salinities of 0.0 to 0.035 kg kg^{-1}.

The solute transport and groundwater flow equations were solved using the finite element code escript[1,2]. We follow an iterative coupling algorithm[10] in which the solute transport equation (equation 2), equations of state (equations 5 and 6) and groundwater flow equations (equations 1 and 4) are solved iteratively until the change in pressure and concentration between two iterations is less than 1×10^{-4} Pa and less than 1×10^{-7} $kg\ kg^{-1}$, respectively.

To assess how well the model can simulate fresh and salt water discharge in coastal aquifer systems we compared the model code with experimental data from a salt water intrusion experiment[11], with analytical solutions of terrestrial groundwater discharge[12,13] and comparison of modeled coastal discharge fluxes with the widely used model code SUTRA[4].

The published salt-water intrusion experiment[11] consists of a tank filled with porous material that is in contact with a column of salt water on one side and a column of fresh water on the other. A hydraulic gradient is imposed from the fresh water side to the salt water side, which causes a net freshwater flow across the model domain. Over time a salt water wedge forms at the salt-water edge of the tank. The position of the salt wedge and the rate of freshwater discharge are strongly affected by the coupling between density-driven flow and solute transport. The experiment is therefore a good benchmark for model codes of coupled flow and solute transport [11], and a better alternative to than the Henry problem [14], which a standard benchmark problem but is less sensitive to the density coupling [15]. The modeled and measured salt wedge position is show in Supplementary Figure 9 for three experiments where different hydraulic gradients were applied. The model results are close to the experimental results, with a mean absolute error of 0.01 m for the position of the fresh-salt water boundary. The modeled position of the fresh-salt water interface is identical to the position

modeled with the widely used model code Sutra [4]. More importantly, the modeled freshwater discharge for the three experiments of 1.41, 0.61 and 1.17 $cm^3 s^{-1}$ is almost identical to the measured fluxes of 1.42, 0.59 and 1.19 $cm^3 s^{-1}$, respectively, which means that the modeled freshwater fluxes are relatively accurate.

The model simulates onshore discharge using a seepage algorithm[12]. The modeled extent of the area where seepage takes place was compared to an analytical solution of the seepage boundary[12], which uses Dupuit assumptions, i.e., the vertical component of groundwater flow is assumed to be zero. The results are shown in Supplementary Figure 10. Our results agree show good agreement with the analytical solution, but consistently show a slightly smaller size of the discharge zone. This is in agreement with numerical experiments by Bresciani et al.[16] who shows that the analytical solution overestimates the size of the seepage zone due to the limitations imposed by the Dupuit assumption.

The modeled discharge fluxes over the land surface were compared to an analytical solution of groundwater discharge by Bokuniewicz[13]. The analytical solution assumes a constant linear hydraulic gradient over the land domain instead of the more realistic recharge and seepage boundary that we used in GroMPy-couple. We adopted a specified hydraulic head boundary condition for three model runs that were compared to the analytical solution. The modeled discharge flux matches the analytical solution perfectly (Supplementary Figure 11).

Finally, modeled fresh and salt water fluxes were also compared to modeled fluxes by Sutra. A comparison of modeled terrestrial and submarine groundwater discharge simulated by GroMPy-couple and Sutra is shown in Supplementary Figure 12. For these model setups the upper boundary condition was simplified to a fixed watertable (i.e., fluid pressure is zero) at the land surface. This is because Sutra does not have the option to simulate a seepage boundary. All other conditions were equal to the model runs that were used to explore submarine groundwater discharge in this manuscript. The results show a good match between Sutra and GroMPy-couple. GroMPy-couple predicts slightly higher recirculated submarine groundwater discharge fluxes, with an average of 12% of the total submarine groundwater discharge being saline in the Sutra model experiments and 15% in GroMPy-couple. The cause for this is unknown. However, because the difference is very small compared to the other uncertainties (such as permeability) in modeling coastal groundwater discharge and both models show identical results in the salt-water intrusion benchmark discussed previously we consider GroMPy-couple sufficiently accurate to quantify coastal groundwater discharge.

Supplementary Note 2

The model runs that were used to calculate the global coastal groundwater fluxes use fixed values of aquifer thickness, permeability anisotropy and dispersivity. The degree to which this may affect model result is shown by results of sensitivity analysis in Supplementary Figure 13. Compared to the much more sensitive parameters topographic gradient and permeability, coastal discharge is relatively insensitive to dispersivity and permeability anisotropy. In contrast, aquifer thickness does affect the discharge fluxes significantly. The adoption of a constant thickness for the global model results may therefore introduce significant uncertainties to the global model. We estimate this uncertainty as roughly a factor two, based on global estimates of the depth of young groundwater and active groundwater flow[17,18]. Note that this source of uncertainty is still much lower than the order of magnitude uncertainty in the global permeability dataset that was used in our analysis.

Supplementary Note 3

The cross-sectional models use a simple linear topographic gradient. The topographic gradient is important because it sets a maximum for the hydraulic gradient in each watershed and because it governs the partitioning between terrestrial groundwater recharge, terrestrial discharge and submarine groundwater discharge. We evaluated two metrics as representative topographic gradient: the average topographic gradient of coastal watersheds and the topographic gradient of drainage features (i.e., streams) only. To explore which metric provides the best model of coastal groundwater discharge we compared a series of numerical model runs which used a 2D map-view model that included the full topography of coastal watersheds with a series of simplified cross-sectional models. The map-view models were conducted using a standard finite difference model code of steady-state depth-integrated groundwater flow implemented in the programming language Python[19] and employed an identical seepage algorithm[12] as described previously for the cross-sectional models to simulate groundwater discharge. The map-view models include the full topography of each raster cell in the elevation dataset, and are therefore expected to provide a better estimate of groundwater recharge and discharge processes than the cross-sectional models. The map-view models simulate depth-integrated groundwater flow in a single layer, which is a common assumption in regional-scale groundwater models. The models used elevation data from a global digital elevation dataset[20], and recharge and permeability following the results of the geospatial analysis described in section S1. We simulated groundwater flow and discharge in 59 randomly chosen watersheds and compared the modeled terrestrial, near-shore and submarine groundwater flux in the map-view model with the fluxes simulated in the cross-sectional models. For

this series of model runs both the map-view and cross-sectional models simulated single-density groundwater flow and did not include solute transport.

The results of a single model run for an example watershed are shown in Supplementary Figure 6. A comparison of the recharge and discharge fluxes as a function of the distance to the coastline for the map-view and the cross-sectional models are shown in Supplementary Figure 14f. In total 36% of the groundwater that is recharged discharges within 500 m of the coastline for the map view model. Groundwater discharge is distributed along topographic lows that coincide with surface water drainage features in the watershed. When projected to the distance from the coastline groundwater recharge and discharge are distributed unevenly (Supplementary Figure 14f). In the cross-sectional models recharge is focused in a small area upstream and there is a large area where recharge equals discharge and the net flux over the top boundary is zero (Supplementary Figure 14f). The cross-sectional model was rerun two times, each time with a different topographic gradient corresponding to the average gradient of the entire watershed and the average gradient of the main drainage channel in the watershed. The modeled discharge for these two cases is 39 and 11% of the applied recharge, respectively. This shows that in this case the cross-sectional model with the average topographic gradient compares best to the modeled discharge in the map-view model.

Comparison of the modeled coastal discharge fluxes for cross-sectional and map-view models for 59 randomly chosen watersheds is shown in Supplementary Figure 15. For 44 out of the 59 watersheds the modeled coastal groundwater discharge as a percentage of the total recharge in the map-view model is covered by the three cross-sectional models or the difference is 10% or less. Overall, the average topographic gradient of the entire watershed and the gradient of the streams are both equally good predictor of coastal groundwater discharge, with a coefficient of determination (R^2) value of 0.51 and 0.50, respectively. While cross-sectional models clearly do not capture the full variation of groundwater recharge and discharge, overall they area a relatively good first order approximation of coastal groundwater discharge, provided that models using the average topographic gradient and the topographic gradient of streams only are both taken into account.

Supplementary Note 4

We compared the modeled groundwater table gradient with a global map of watertable gradients that is based on a global compilation and model of watertable depths[21]. Note that the modeled watertable gradients were calculated by dividing the hydraulic head at the right-hand side of the model domain by the size of each model. In total 336 coastal watersheds had one or more observation of watertable depth in the global database. The comparison of the modeled and observed average watertable in these watersheds shows a reasonable agreement (Supplementary

Figure 6). The median ratio is 1.06, which means that our model may slightly overestimate watertable gradients and underestimate permeability and coastal groundwater discharge. The model explains slightly less than half the observed variance in watertable gradients. The coefficient of determination (R^2) is 0.57, which suggest that while overall the model matches the gradients well it does not fully capture local variability and should be considered as a first order estimate that is representative for relatively large spatial scales (i.e., watershed size, ~11 km). While a calibration of the coastal groundwater discharge model to match local watertable data would be beyond the scope of this study, the agreement between the mean modeled and observed watertables indicates that the model results presented here are relatively robust.

The modeled values of coastal groundwater discharge and fresh SGD were compared to local fresh SGD estimates. While there are abundant estimates of total SGD in the literature studies that provide a robust quantitative estimate of the contributions of the fresh component of SGD are relatively scarce. We compiled fresh SGD estimates that were predominantly obtained using seepage meters, where the fresh component of SGD was estimated using a salinity balance of the water discharging in seepage meters[22–26] or direct sampling of discharge[27]. In addition, a number of estimates were based on a combination of seepage meters and radon or radium isotopes in seawater[28,29], seawater salinity anomalies to estimate the fresh water contribution[30], and pore water chemistry and models of solute flux in the seabed[31]. We did not include the relatively numerous estimates of fresh SGD that were calculated using Darcy's law, because these implicitly assume that all water flowing in coastal aquifers discharges directly in the ocean, do not take into account the interaction of fresh and salt water at the coastline and diverge strongly from analytical models[13,32] or numerical models of coastal groundwater discharge like the model presented here.

Comparison of modeled and reported coastal groundwater discharge values in Supplementary Figure 16a and Table 6 shows that the modeled discharge is in several cases much lower than the values reported in many locations. Note that the reported values of fresh SGD were compared to modeled CGD and not modeled fresh SGD because especially for the studies that did not use seepage meters the reported fresh SGD may include discharge above the mean sea level and below the high tide line, and because the modeled CGD is a more robust number than the contributions of modeled fresh SGD or NGD due to the sensitivity of the partitioning of onshore and offshore discharge to near-shore topographic gradients. If only modelled fresh SGD would be used the values for the modelled fluxes in Supplementary Figure 16a would be half or less of the values that are shown. Five studies[22,26,27,29,31] report values that are in the uncertainty range presented by our model results. The remaining five studies[23–25,28,30] show values that are one or more orders of magnitude higher than the model predictions. However, comparison of modeled values of

permeability and reported values by five of these studies (see Table 6) show that with the exception of one study these values are in the same order of magnitude, and the misfit with reported fresh SGD values is not likely to be due to a systematic underestimation of permeability in our models.

Reported values of fresh submarine groundwater discharge are often equal or higher than the total recharge input in the system (Supplementary Figure 16b). All the fresh SGD estimates are from locations that have mapped rivers in the hinterland, which normally channel substantial part of the overall recharge in coastal aquifer as river baseflow and which means that only a small part of recharged groundwater can contribute to submarine groundwater discharge. Note that the reported fresh SGD values are from humid settings where models of groundwater recharge[33,34] are relatively reliable[35,36]. The contradiction between reported submarine groundwater discharge rates and the amount of groundwater available in coastal watersheds to supply fresh SGD has been noted by several previous authors[37,38]. The reasons for the mismatch may be the difficulty and high uncertainty of separating the fresh and recirculated components of SGD in local studies[39,40], and potential bias of sampling locations towards sites of relatively high and focused discharge that is visible at the shoreline[41]. Furthermore, to our knowledge sites with low or no SGD tend to not get reported in the literature.

Initial estimates of coastal discharge assigned a permeability of $10^{-11.8}$ m^2 to carbonate units following the global permeability map. The resulting estimates of coastal discharge were lower than expected for coastal karstic aquifers. For instance in the Yucatan peninsula surface water features are absent, and the majority of groundwater recharged is expected to discharge near the coast, along karst conduits and coastal and submarine springs[42]. Initial model runs predicted a coastal discharge of only 20% of the groundwater recharge for the eastern part of Yucatan. The modified permeability values where a higher permeability was assigned to carbonate units resulted in near-shore discharge that was equal to 87% of the groundwater recharged in the coastal watershed. This value is in accordance with discharge estimates for this region[42]. However, the total flux may still be underestimated, comparison with stream locations on Google Earth suggests that while overall the watershed and stream database[43] that support our analysis is robust, in areas with extensive karst such as Yucatan the stream density may be overestimated, which means that the representative length scale and the total groundwater input may be underestimated in our models. However, areas with continuous karstic carbonate cover over scales that exceed the scales of coastal watersheds (~11 km) are rare globally[44].

A comparison of modeled fresh SGD and coastal groundwater discharge with nine locations where significant use of fresh submarine groundwater has been reported[45] is shown in Table 1. In addition,

a selection of seven locations that have reported strong impacts of fresh SGD on coastal ecosystems is shown in Table 2. The latter is admittedly a small sample of the large body of literature on the impacts of fresh SGD, a more extensive compilation is planned as a follow up study. In most locations that report use of fresh SGD the modeled fresh SGD exceeds 50 m^2 a^{-1} (Table 1), which is much higher than the global median discharge of 0.4 m^2 a^{-1}. In two locations (Bahrain and Quissico, Mozambique) the modeled fresh SGD is very low. However, in both cases the modeled total coastal groundwater discharge is much higher (50 and 233 m^2a^{-1}, respectively) than the fresh SGD component. Since the partitioning between fresh SGD and near-shore terrestrial discharge is highly dependent on topographic gradient the underestimation is likely due to the models not representing coastal topography well enough by using a single linear topographic gradient.

The coastal (onshore and offshore) groundwater discharge in locations where effects on ecosystems have been reported (Table 2) is in most cases much higher than the median value of 30 m^2a^{-1}. The values of nitrogen application in the adjacent coastal watersheds are highly variable and in some cases relatively low. This suggests that either other sources of nutrients or pollutants affect the coastal ecosystems or that agricultural nitrogen application has been underestimated in the relatively low-resolution global dataset that was used[46]. In addition, in these local studies it may have been in some cases difficult to separate the effects of surface water and groundwater input. Nonetheless overall the model results successfully match the relatively high discharges required to explain locations with significant use of fresh SGD or where coastal groundwater discharge impact the solute and nutrient budgets of coastal ecosystems.

Supplementary Figures

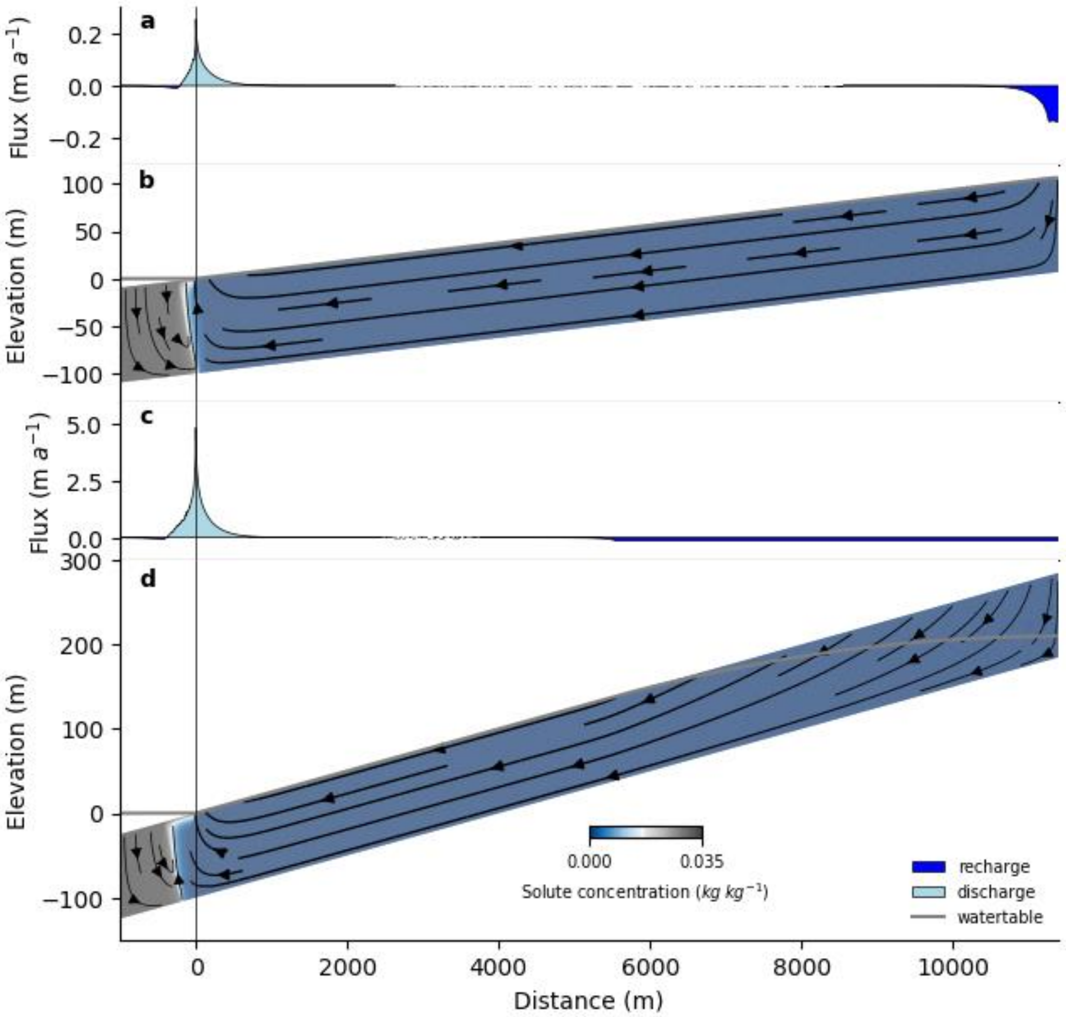

Supplementary Figure 1. Examples of modeled groundwater flow, salinity and distribution of terrestrial and submarine recharge and discharge. The results are shown for two different model runs, one for a model run that uses the median global watershed (panels a and b) and a second model run that used a relatively high permeability (10^{-12} m^2) and topographic gradient (2.5%) (panels c and d). The flux over the top surface (i.e., the land surface and the seabed) is shown in panel a and c, and shows the distribution of areas with groundwater recharge (dark blue), areas with zero or near-zero discharge where the applied recharge is balanced by discharge and a relatively small zone with significant terrestrial and submarine groundwater discharge (light blue).

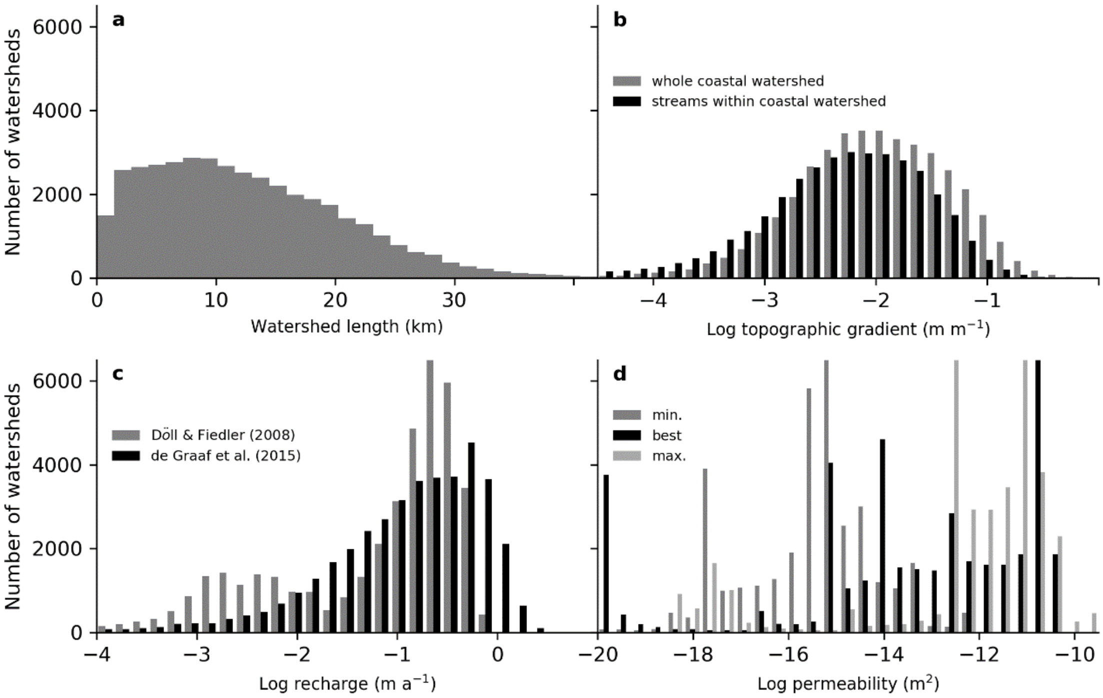

Supplementary Figure 2. Results of the global geospatial data analysis of hydrogeological parameters in coastal watersheds. The figure shows the representative watershed length (panel a), topographic gradient of coastal watersheds and streams in these watersheds (panel b), groundwater recharge in coastal watersheds (panel c) and average permeability in coastal watersheds (panel d). The min. and max. estimates of permeability take into account the 1σ uncertainty range reported by Gleeson et al. [47,48].

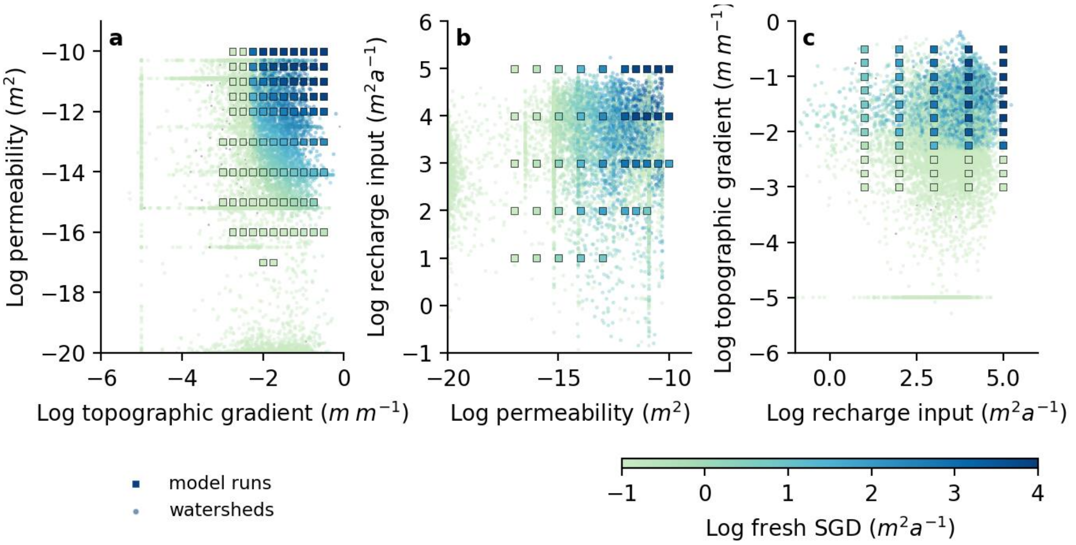

Supplementary Figure 3. Illustration of the linear interpolation method used to quantify fresh submarine groundwater discharge at a global scale. This figure shows the interpolation of fresh submarine groundwater discharge (SGD) for each of the 40082 global coastal watersheds from a series of 351 model runs that cover the range of permeability, topographic gradients and groundwater recharge found in these watersheds. Each dot represents the parameter values for permeability, topographic gradient and recharge volume for a single coastal watershed, which were derived from a global geospatial analysis (see Methods). The watersheds are colored by their interpolated value of fresh SGD. Each square represents a single numerical model run. Each panel shows the parameter values and interpolated fresh SGD for a pair of parameters; permeability and topographic gradient (panel a), permeability and recharge volume (panel b) and recharge volume and topographic gradient (panel c). Note that in each panel there are a number of model runs (squares) that overlap. In each case the model run is shown with the highest modeled fresh SGD. For instance, for panel a behind each square there are a number of additional model runs that are not shown that used different recharge volume values. The results of the model experiments can be found in Supplementary Data 2. The geospatial data of coastal watersheds and the interpolated values of coastal groundwater discharge are available as Supplementary Data 3.

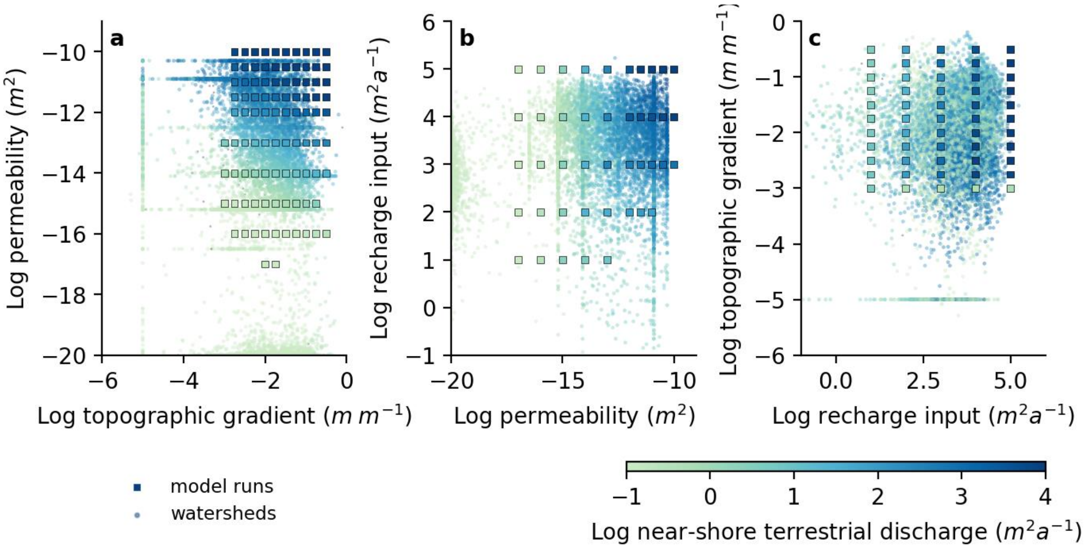

Supplementary Figure 4. Interpolation of near-shore terrestrial groundwater discharge (NGD) for each of the n=40082 coastal watersheds in our global database from n=351 model runs.

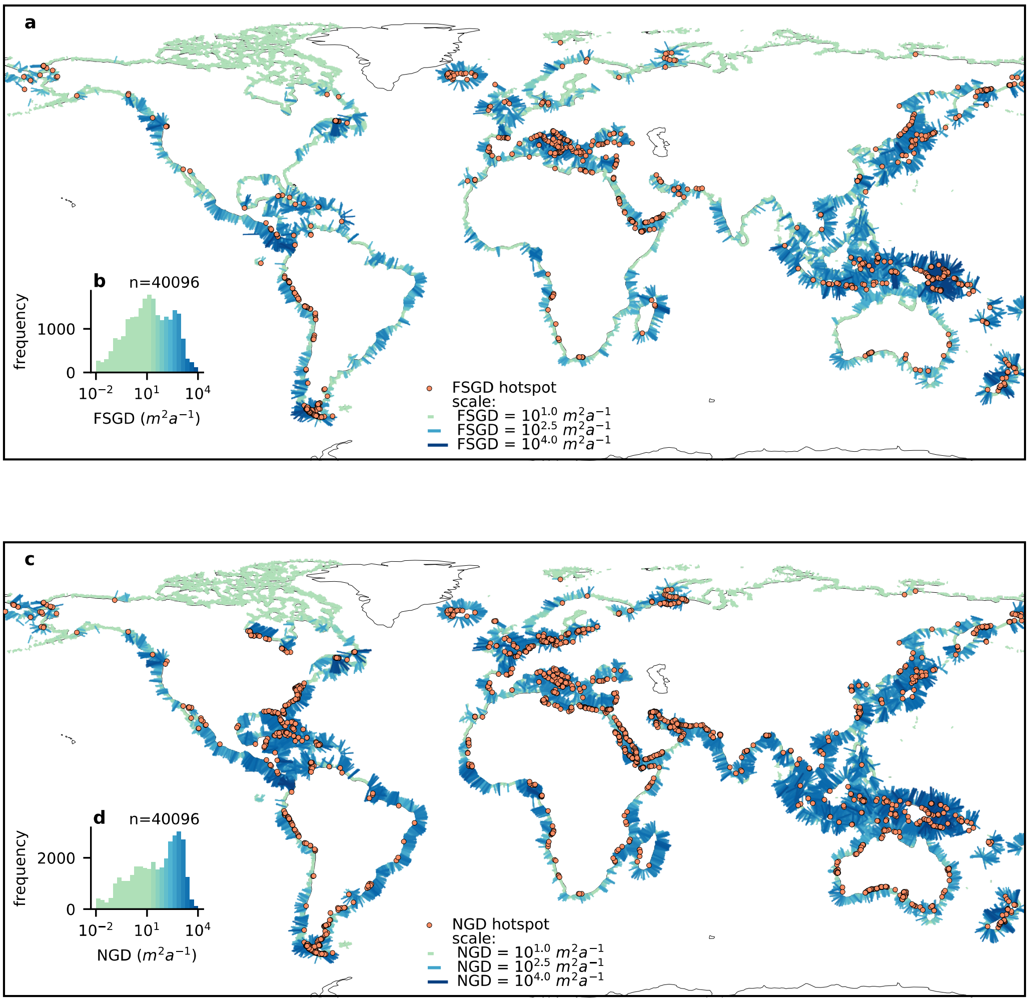

Supplementary Figure 5. Modeled global fresh submarine groundwater discharge (SGD) (panel a) and near-shore terrestrial groundwater discharge (NGD) (panel b). Hotspots denote locations where the discharge flux exceeds 100 m^2a^{-1} and 25% of the surface water flux to the oceans. Fresh SGD and NGD were calculated for each of the n=40082 coastal watersheds by linear interpolation of n=351 model runs. See Supplementary Figure 3 and 4 and the Methods for more information.

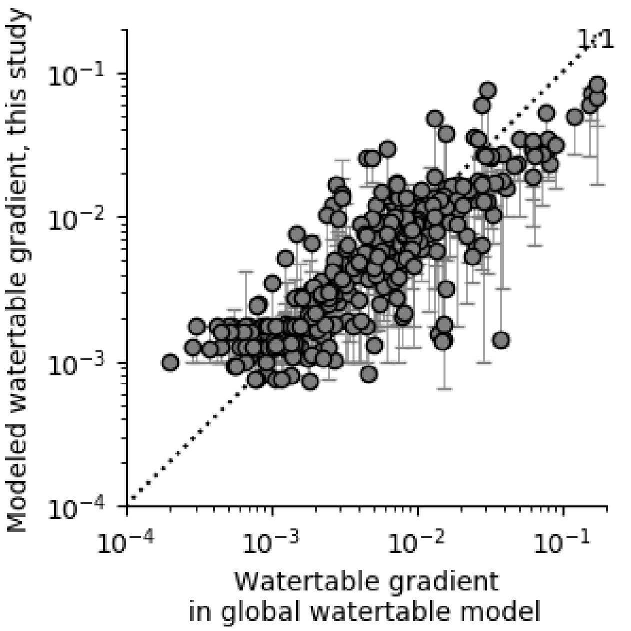

Supplementary Figure 6. Comparison of the modeled watertable gradient of this study with the watertable gradient from a published global data compilation and model[21] for 336 coastal watersheds with at least one watertable datapoint.

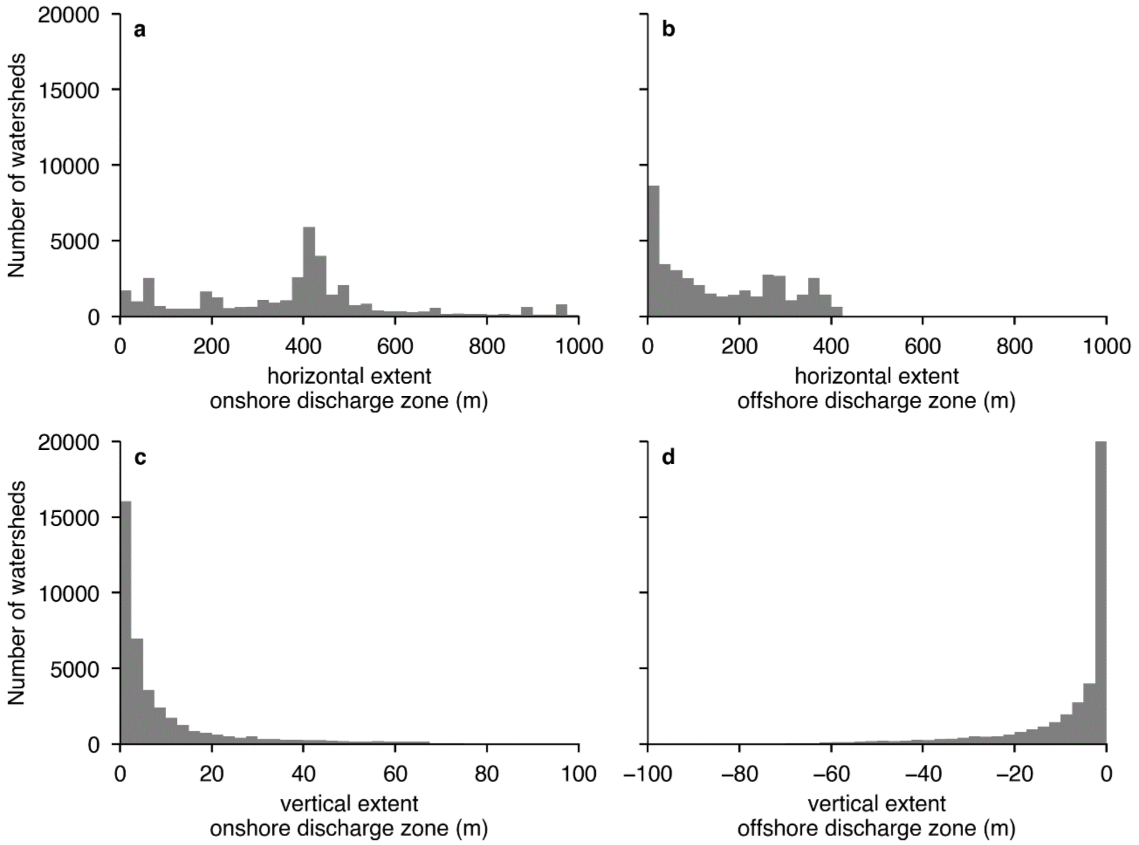

Supplementary Figure 7. Distribution of the extent (a, b) and elevation (c, d) of the modeled terrestrial (a, c) and submarine (b, d) coastal groundwater discharge for each coastal watershed. Note that these values are relatively uncertain due to the strong effect of permeability anisotropy and aquifer thickness on the location of the discharge zone, which are poorly constrained parameters in our analysis. The size of the discharge zone is defined as the area where 90% of all groundwater discharge takes place. Note that this does not include areas where discharge is equal or lower than the applied rate of groundwater recharge.

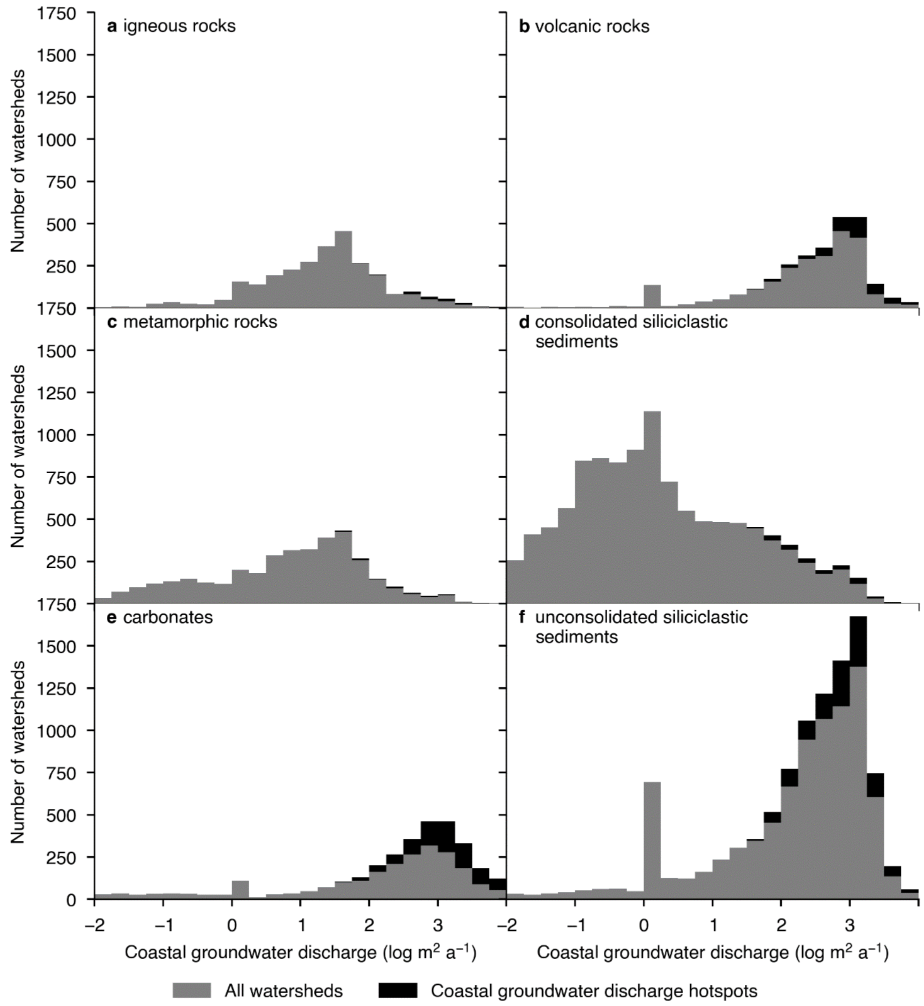

Supplementary Figure 8. The distribution of modeled coastal groundwater discharge and coastal groundwater discharge hotspots for different lithologies based on the global lithology map[44]. Model results predict that coastal groundwater discharge predominantly takes place in coastal watersheds consisting of permeable volcanic (b), carbonate (c) or unconsolidated siliciclastic rocks (f).

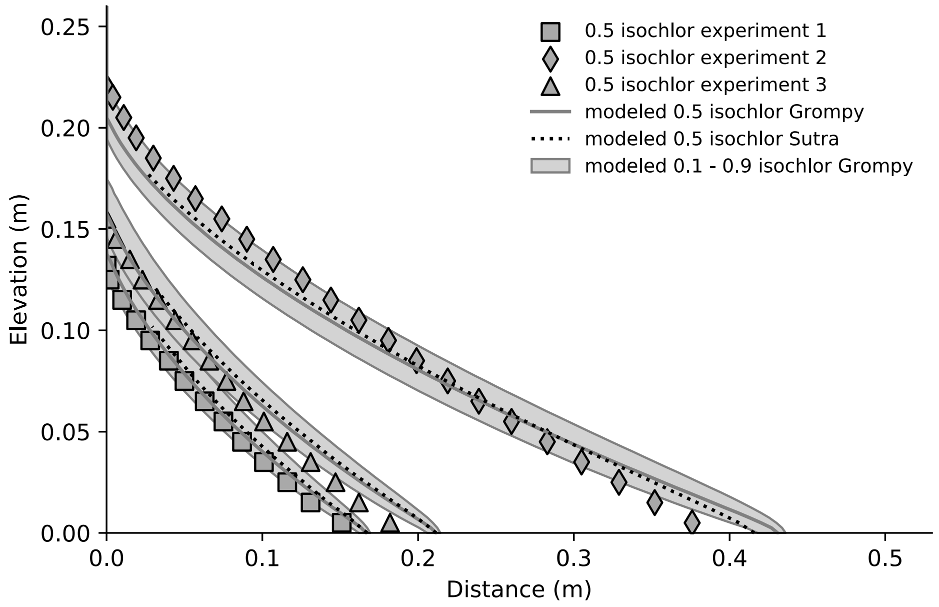

Supplementary Figure 9. Comparison of modeled position of a salt wedge by GroMPy-couple to the measured position in fresh and salt water flow experiments in porous media[11]. The modeled position of the fresh-salt water interface compares well to the measured values and is comparable to results for the widely used model code SUTRA[4]. The position of the salt wedge is equal to the location of the 50% salt water concentration contour line (0.5 isochlor).

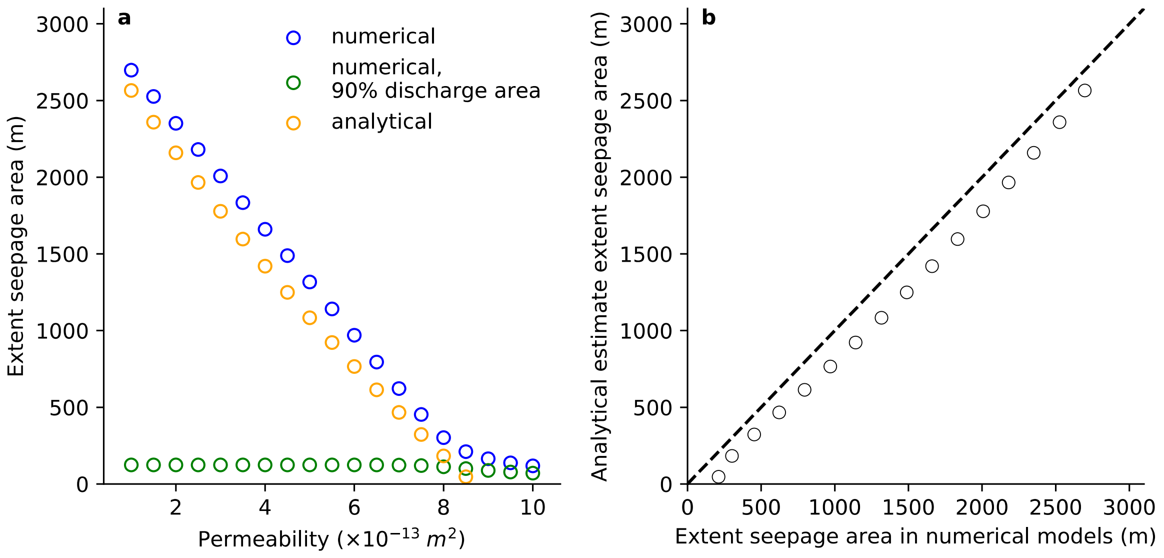

Supplementary Figure 10: Comparison of numerical estimates of the size of the onshore discharge zone with an analytical solution [12]. Panel a shows a comparison of the size of the discharge zone and permeability in a series of 20 model runs. Panel b shows the size of the seepage zone in numerical models and the analytical solution. The results show good agreement between the models and the analytical solution, and confirm the moderate underestimation of seepage zones by the analytical solution reported by Bresciani et al.[16] While the seepage zone is relatively large in most model experiments, the majority of onshore discharge is concentrated near the shoreline as shown by the much smaller area where 90% of the onshore discharge takes place in panel (a). The model runs shown here used a domain length of 3000 m, a thickness of 100 m, a recharge flux of 0.1 m a^{-1} and a topographic gradient of 0.01.

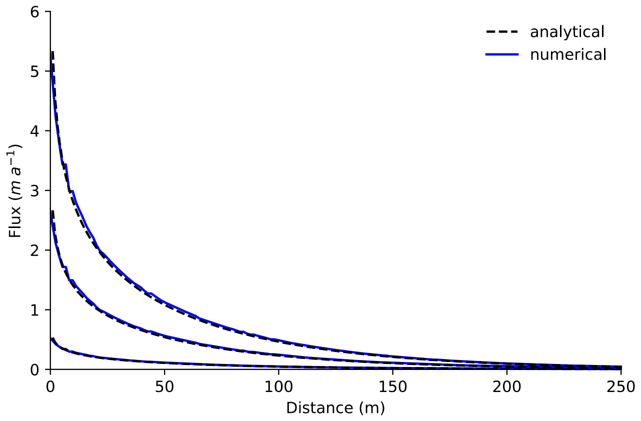

Supplementary Figure 11: Comparison of modeled groundwater discharge and calculated discharge using an analytical solution [13]. The model experiments shown used a specified pressure of 0 Pa at the entire land surface, a topographic gradient of 0.01, aquifer thickness of 100.0 m and a isotropic permeability of 10^{-13} m^2, 5.0 $\times 10^{-12}$ m^2 and 1×10^{-12} m^2.

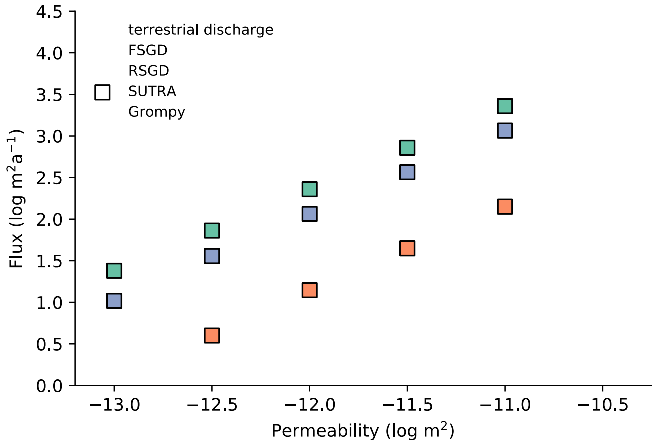

Supplementary Figure 12. Comparison of modeled fresh and recirculated submarine groundwater discharge and terrestrial discharge in a series of identical model experiments with the model codes GroMPy-couple and SUTRA. The model experiments used model domain size of 2.5 km onshore and 500 m offshore, a topographic gradient of 0.01 and a thickness of 100 m. In contrast to the remaining model runs in this study the upper boundary did not consist of a mixed seepage and recharge boundary, but used a specified pressure of 0 Pa at the land surface.

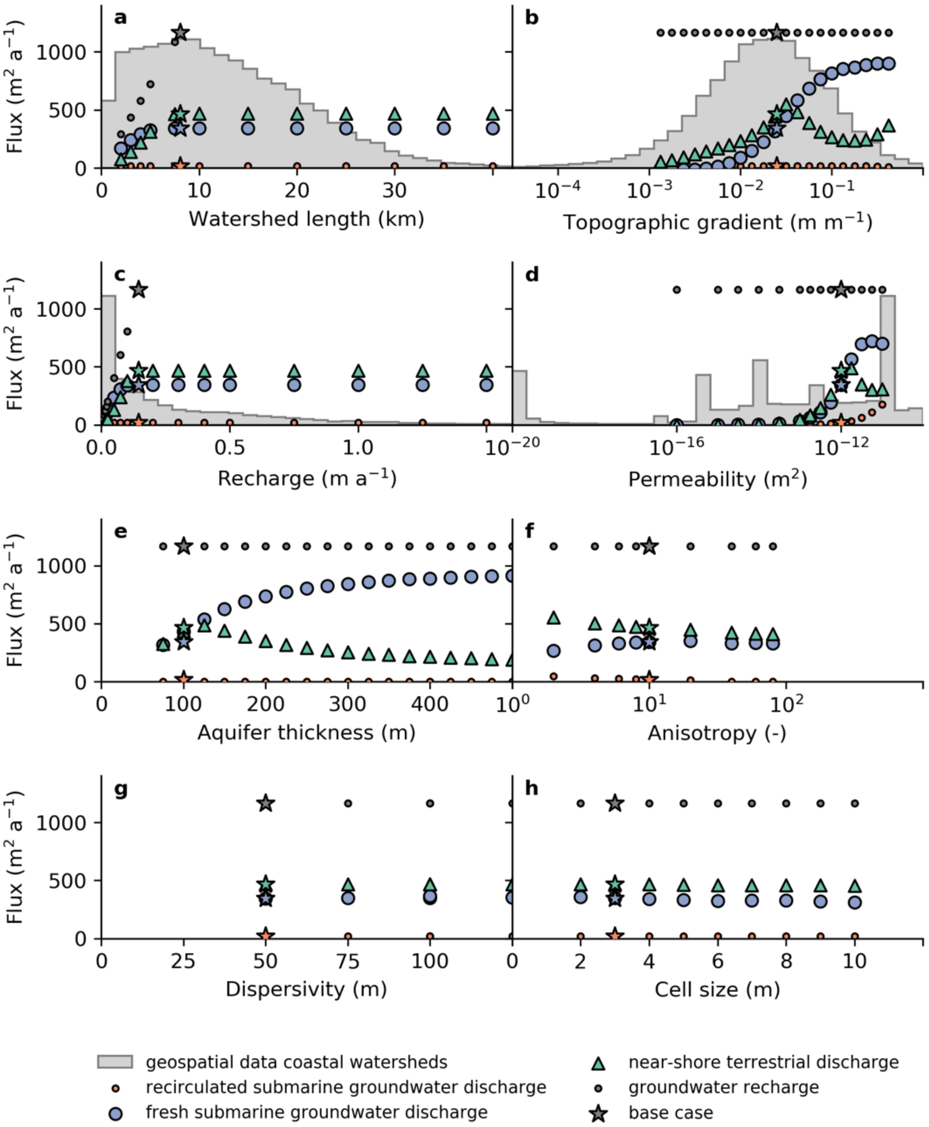

Supplementary Figure 13. Sensitivity of coastal groundwater discharge to watershed length (a) topographic gradient (b), groundwater recharge (c), permeability (d), aquifer thickness (e), permeability anisotropy (f), longitudinal dispersivity (g) and grid cell size (h).

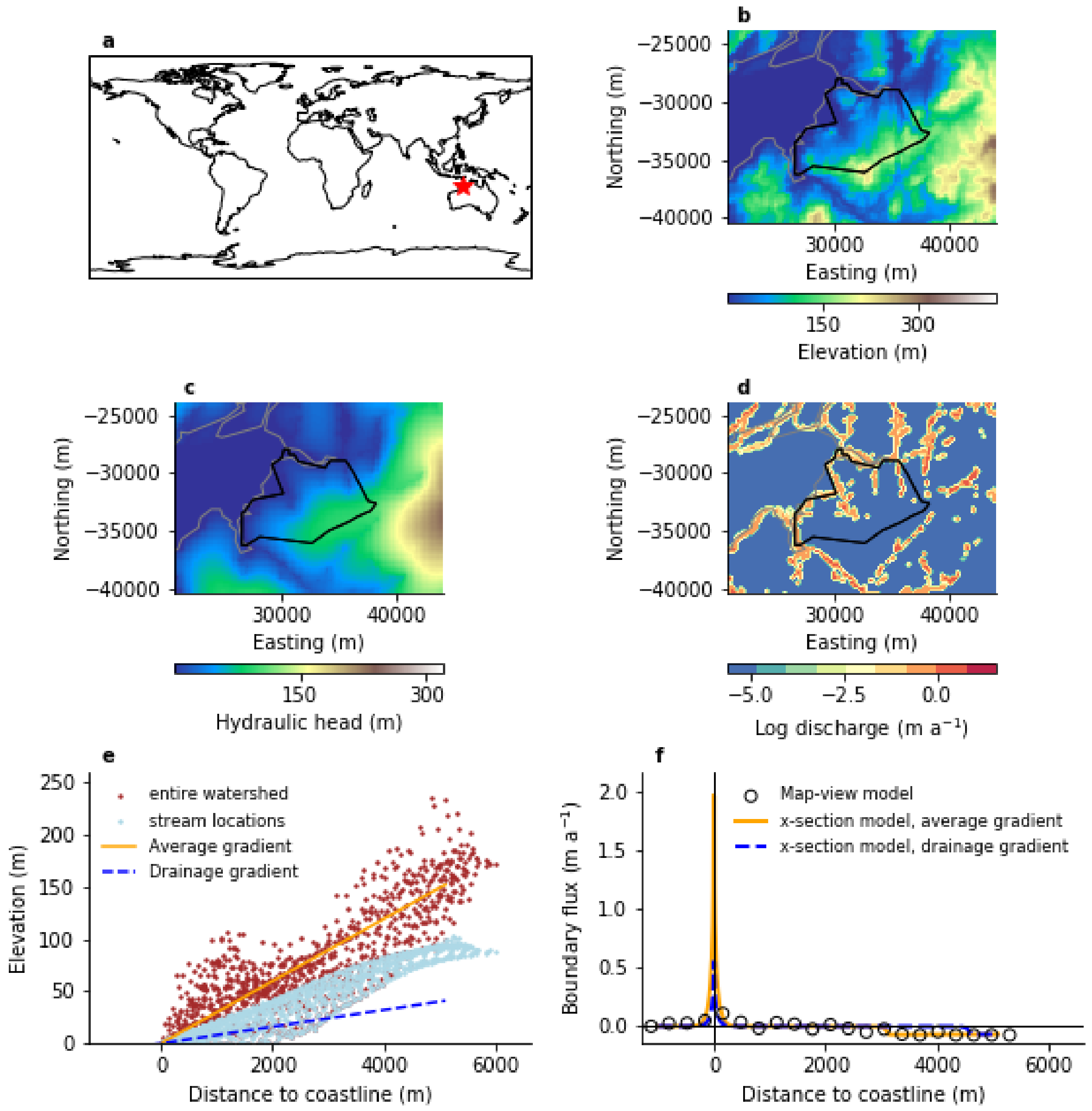

Supplementary Figure 14. Comparison of coastal groundwater flow and discharge in map-view and cross-sectional models for an example coastal aquifer in Australia. Panel a shows the location of the watershed. Panel b, c and d show elevation, modeled hydraulic head and modeled groundwater discharge in the map-view model. Panel e shows the elevation of the entire coastal watershed and the streams in the watershed along with two regression lines for elevation vs distance to the coast. Panel f shows a comparison of the modeled groundwater discharge in the map-view model and cross-section models that use the average topography or the average topographic gradient of the streams. Overall the map-view model predicts a coastal discharge of 36% of the total recharge, and the cross-sectional model predict 39% when using the average topographic gradient and 11% when using the gradient of the stream in the coastal watershed.

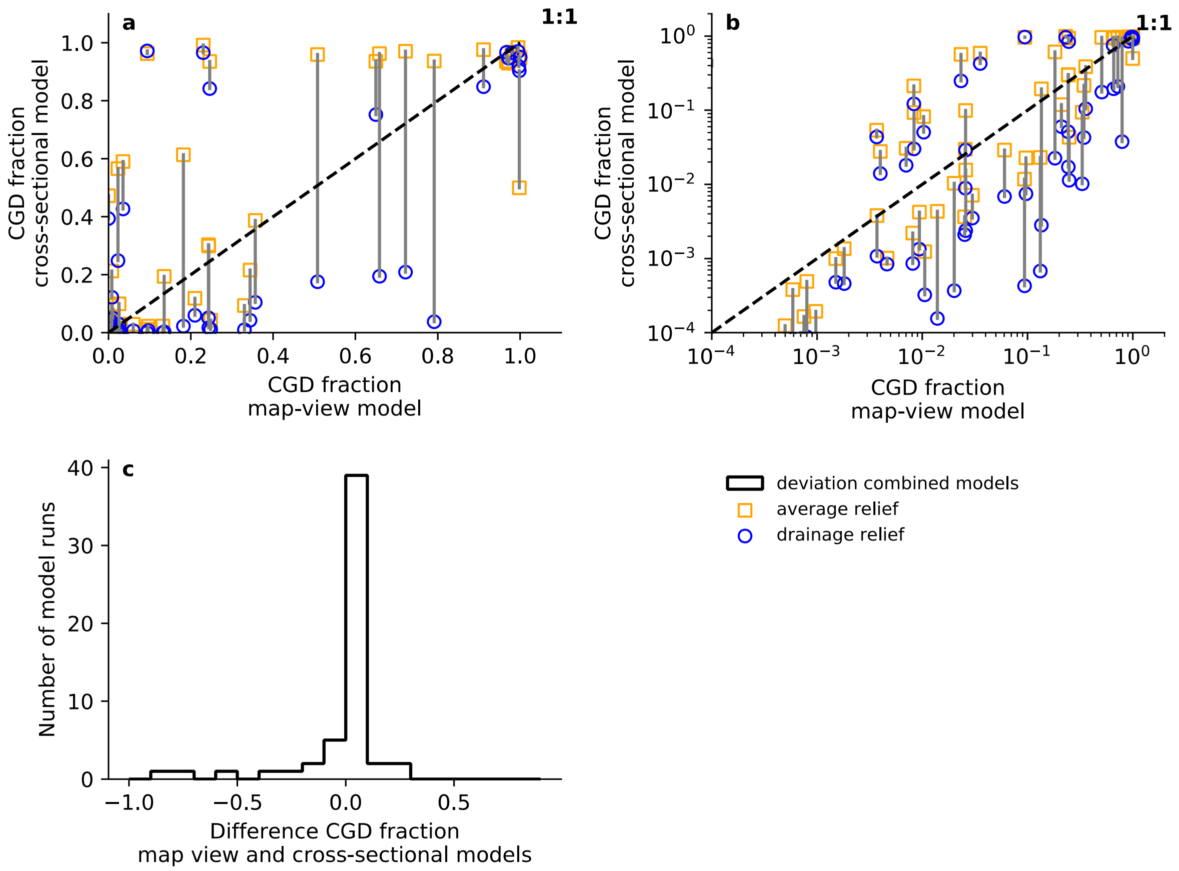

Supplementary Figure 15. Comparison of coastal groundwater flow and discharge in map-view models and cross-sectional model for 59 randomly chosen coastal watersheds. The majority of watersheds groundwater discharge can be approximated well in the 2D cross-sectional models by using either the average topographic gradient or the average gradient of the stream nodes in each watershed. For 75% of the watersheds the cross-sectional discharge falls between these values or the difference between the modeled discharge is less than 10%. Panel a and b show the modelled CCD fraction in the map-view model and the cross sectional model. Panel b shows the same results as panel a, but in t logarithmic scale. The difference in CGD fraction between the two model approaches is summarized in panel c.

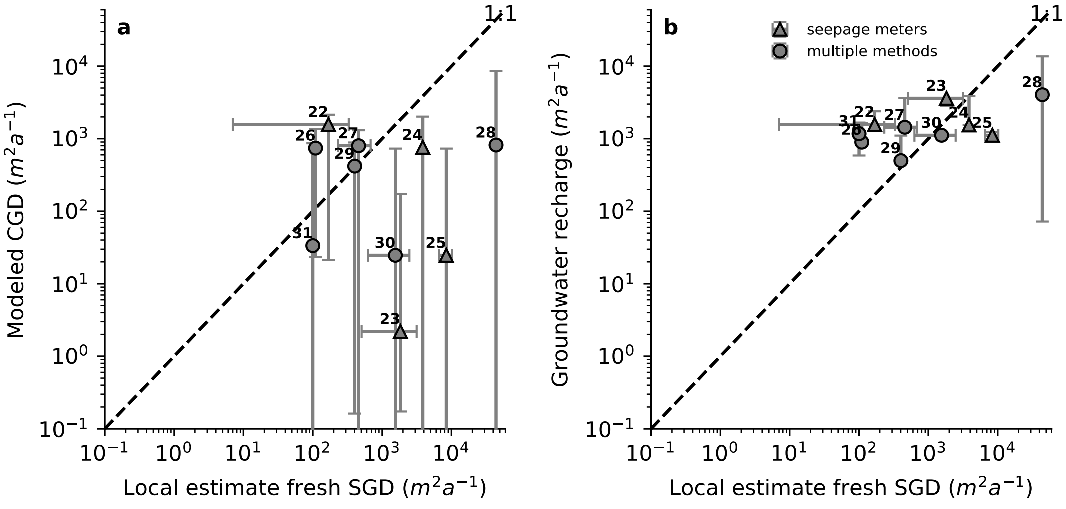

Supplementary Figure 16. Comparison of reported fresh SGD and modeled coastal groundwater discharge and groundwater recharge for 10 selected case studies. Five out of ten reported fresh SGD estimates are much higher than our model results (panel a), but these estimates are also equal to or exceed the total freshwater input in adjacent coastal aquifers (panel b). Numbers denote the reference for each estimate.

Supplementary Table 1. Modeled coastal groundwater discharge for locations with reported use of coastal groundwater[45].

**Country**	**Location**	**Reported use of SGD**	**Modeled fresh SGD (m^2 a^{-1})**		
			**best**	**min.**	**max.**
Syria	Latakia	Collected by boat from springs 4km offshore	106	0.004	281
Lebanon	Chekka	Water collected by plastic tubing until the 1960s	768	11	583
Bahrein		Drinking water	5	0.0	5
Indonesia	Ngobaran Beach, Java	Drinking water	2354	38	2354
Greece	Kiveri	Submarine spring dammed and used for irrigation	94	15	123
Peru	Surquillo	Drinking water	600	0.4	855
Mozambique	Quissico	Washing	0	0.0	138
Indonesia	East Lombok	Drinking water	691	4.9	1733
Australia	Pt. Willunga	Submarine springs important for local aboriginal culture	76	0.0	52

Supplementary Table 2. Modeled coastal groundwater discharge for locations with reported impacts of groundwater discharge on coastal solute and nutrient budgets.

**Reference**	**Country**	**Location**	**Reported impact of coastal groundwater discharge**	**Modeled CGD (m^2 a^{-1})**			**Nitrogen application**
				best	min.	max.	kg ha^{-1}
[26]	Portugal	Ria Formosa	Eutrophication	546	26	989	8
[42]	Mexico	Eastern Yucatan	Pollution of mangroves and coral reefs	92	0.1	308	0.2
[49]	Israel	Eilat	Eutrophication coral reef	49	0.2	62	1.0
[49]	U.S.A.	Key Largo	Eutrophication coral reef	394	0.2	2480	0.9
[50]	Australia	Moreton Bay	Carbon budget mangroves	233	0.03	818	21
[51]	U.S.A.	South Carolina	Nutrient budget salt marsh	177	0.2	315	2
[52]	Australia	Perth	Nutrient budget lagoon	160	11	181	22
[53]	U.S.A.	Tampa Bay	Algal blooms	766	0.01	2019	56

Supplementary Table 3. Statistics for hydrogeological variables in the 40,082 global coastal watersheds derived from global geospatial data analysis.

Variable	Units	Median	Average	Standard deviation
Watershed size	*km*	11.4	12.6	8.2
Topographic gradient, all raster cells	$m\ m^{-1}$	0.0094	0.025	0.043
Topographic gradient, streams	$m\ m^{-1}$	0.0050	0.013	0.024
Recharge [33]	$m\ a^{-1}$	0.132	0.167	0.164
Recharge [34]	$m\ a^{-1}$	0.143	0.289	0.360
Log-transformed permeability, minimum estimate	m^2	-15.4	-16.1	2.4
Log-transformed permeability, best estimate	m^2	-12.9	-13.5	2.7
Log-transformed permeability, maximum estimate	m^2	-11.7	-12.3	2.1

Supplementary Table 4. Parameters ranges used for model sensitivity analysis.

**Parameter**	**Base case value**	**Sensitivity analysis values**
Watershed length (m)	11400	1000, 2000, 3000, 4000, 5000, 7500, 10000, 15000, 20000, 25000, 30000, 35000, 40000
Groundwater recharge (m a^{-1})	0.143	0.001, 0.0025, 0.005, 0.01, 0.015, 0.02, 0.025, 0.05, 0.075, 0.1, 0.2, 0.3, 0.4, 0.5, 0.75, 1.0, 1.25, 1.5, 1.75
Permeability, *k* (log m^2)	-12	-20, -18, -17, -16, -15, -14.5, -14, -13.5, -13, -12.75, -12.5, -12.25, -12, -11.75, -11.5, -11.25, -11, -10.75, -10.5, -10.25, -10.0
Topographic gradient (log m m^{-1})	-1.6	-4 to -0.5, interval = 0.125
Aquifer thickness (m)	100	25-500, interval=25
Permeability anisotropy, (dimensionless)	10	1, 2, 4, 6, 8, 10, 20, 40, 60, 80, 100, 200
Longitudinal dispersivity, α_L (m)	50	25 to 150, interval = 25
Grid cell size (m)	3	1 to 10, interval = 1

Supplementary Table 5. Parameters ranges used for the exploration of parameter space and quantifying global coastal groundwater discharge.

Parameter	Values
Recharge input (log $m^2 a^{-1}$)	1.0 to 5.0, interval=1.0
Permeability (log m^2)	-16, -14, -13, -12, -11.5, -11, -10.5, -10
Topographic gradient (log m m^{-1})	-3 to -0.5, interval=0.25

Supplementary Table 6. Comparison of modeled coastal groundwater discharge and published local estimates.

Reference	Country	Location	Reported fresh SGD ($m^2 a^{-1}$)			Method	Modeled CGD ($m^2 a^{-1}$)			Reported permeability (log m^2)	Modeled permeability (log m^2)
			best	min.	max.		best	min.	max.		
[30]	U.S.A	Stinson Beach, California	1552	631	2472	Salinity budget seawater	24.7	0.1	732	-10.3	-14.0
[27]	U.S.A.	Delaware	458	231	684	Direct sampling of discharge at low tide	800	0.1	1307	n/a	-10.9
[26]	Portugal	Ria Formosa	110			Seepage meters	743	24	1369	n/a	-11.1
[22]	U.S.A	Florida river lagoon	168	7	329	Seepage meters	1571	21	2144	-11.7	-10.3
[23]	U.S.A.	Waquit bay	1830	505	3156	Seepage meters	2	0.2	173	n/a	-14.1
[28]	Brazil	Paros Lagoon	44057			Radium and salinity budget	819	0.1	8623	n/a	-11.2
[24]	U.S.A	Indian River Bay, Delaware	511			Seepage meters	762	0.1	2011	-11.1	-10.9
[29]	U.S.A	northeastern Gulf of Mexico	402			Radon and seepage meters	418	0.2	887	-11.7	-10.9
[31]	U.S.A	San Francisco Bay, California	100			Seepage meter and porewater chemistry model	34	0.01	866	n/a	-13.6
[25]	China	Yellow River Delta	8401	6575	10227	Seepage meters	25	0.1	732	-13.6	-14.0

Supplementary References

1. Gross, L., Bourgouin, L., Hale, A. J. & Muhlhaus, H.-B. Interface Modeling in Incompressible Media using Level Sets in Escript. *Phys. Earth Planet. Inter.* **163**, 23–34 (2007).
2. Poulet, T., Gross, L., Georgiev, D. & Cleverley, J. escript-RT: Reactive transport simulation in Python using escript. *Comput. \& Geosci.* **45**, 168–176 (2012).
3. Langevin, C. D., Thorne Jr., D. T., Dausman, A. M., Sukop, M. C. & Guo, W. *SEAWAT Version 4: A Computer Program for Simulation of Multi-Species Solute and Heat Transport*. (2008).
4. Voss, C. I. & Provost, A. M. *SUTRA, A model for saturated-unsaturated variable-density ground-water flow with solute or energy transport*. (2002).
5. Poulet, T. *et al.* Thermal-mechanical-hydrological-chemical simulations using escript, Abaqus and WinGibbs. in *Abstract for the GeoMod 2010 Conference, 27e29 September* (2010).
6. Hale, A. J. *et al.* Dynamics of slab tear faults: Insights from numerical modelling. *Tectonophysics* **483**, 58–70 (2010).
7. Ingebritsen, S. E., Sanford, W. E. & Neuzil, C. E. *Groundwater in geologic processes*. (Cambridge Univ Press, 2006).
8. Scheidegger, A. E. General theory of dispersion in porous media. *J. Geophys. Res.* **66**, 3273–3278 (1961).
9. Batzle, M. & Wang, Z. Seismic properties of pore fluids. *Geophysics* **57**, 1396–1408 (1992).
10. Ackerer, P. A new coupling algorithm for density-driven flow in porous media. *Geophys. Res. Lett.* **31**, L12506 (2004).
11. Goswami, R. R. & Clement, T. P. Laboratory-scale investigation of saltwater intrusion dynamics. *Water Resour. Res.* **43**, W04418 (2007).
12. Batelaan, O. & De Smedt, F. SEEPAGE, a new MODFLOW DRAIN package. *Ground Water* **42**, 576–88 (2004).
13. Bokuniewicz, H. J. Analytical Descriptions of Subaqueous Groundwater Seepage. *Estuaries* **15**, 458 (1992).
14. Henry, H. R. Salt intrusion into fresh-water aquifers. *J. Geophys. Res.* **64**, 1911–1919 (1959).
15. Simpson, M. J. & Clement, T. P. Theoretical analysis of the worthiness of Henry and Elder problems as benchmarks of density-dependent groundwater flow models. *Adv. Water Resour.* **26**, 17–31 (2003).
16. Bresciani, E., Davy, P. & De Dreuzy, J. R. Is the Dupuit assumption suitable for predicting the groundwater seepage area in hillslopes? *Water Resour. Res.* **50**, 2394–2406 (2014).
17. Gleeson, T., Befus, K. M., Jasechko, S., Luijendijk, E. & Cardenas, M. B. The global volume and distribution of modern groundwater. *Nat. Geosci.* **9**, 161–167 (2016).

18. Jasechko, S. *et al.* Global aquifers dominated by fossil groundwaters but wells vulnerable to modern contamination. *Nat. Geosci.* **10**, 425–429 (2017).
19. Olsthoorn, T. Finite Difference Grounwater Modeling in Python. https://olsthoorn.readthedocs.io/en/latest/01_Numerical_grw_modeling.html# (2017).
20. Danielson, J. J. & Gesch, D. B. Global multi-resolution terrain elevation data 2010 (GMTED2010). *US Geol. Surv. Open File Rep* **1073**, 25 (2011).
21. Fan, Y., Li, H. & Miguez-Macho, G. Global patterns of groundwater table depth. *Science.* **339**, 940–943 (2013).
22. Martin, J. B., Cable, J. E., Smith, C., Roy, M. & Cherrier, J. Magnitudes of submarine groundwater discharge from marine and terrestrial sources: Indian River Lagoon, Florida. *Water Resour. Res.* **43**, (2007).
23. Michael, H. A., Lubetsky, J. S. & Harvey, C. F. Characterizing submarine groundwater discharge: A seepage meter study in Waquoit Bay, Massachusetts. *Geophys. Res. Lett.* **30**, (2003).
24. Russoniello, C. J. *et al.* Geologic effects on groundwater salinity and discharge into an estuary. *J. Hydrol.* **498**, 1–12 (2013).
25. Taniguchi, M. *et al.* Submarine groundwater discharge from the Yellow River Delta to the Bohai Sea, China. *J. Geophys. Res. Ocean.* **113**, (2008).
26. Leote, C., Ibánhez, J. S. & Rocha, C. Submarine groundwater discharge as a nitrogen source to the Ria Formosa studied with seepage meters. *Biogeochemistry* **88**, 185–194 (2008).
27. Hays, R. L. & Ullman, W. J. Direct determination of total and fresh groundwater discharge and nutrient loads from a sandy beachface at low tide (Cape Henlopen, Delaware). *Limnol. Oceanogr.* **52**, 240–247 (2007).
28. Niencheski, L. F. H., Windom, H. L., Moore, W. S. & Jahnke, R. A. Submarine groundwater discharge of nutrients to the ocean along a coastal lagoon barrier, Southern Brazil. *Mar. Chem.* **106**, 546–561 (2007).
29. Santos, I. R., Burnett, W. C., Chanton, J., Dimova, N. & Peterson, R. N. Land or ocean?: Assessing the driving forces of submarine groundwater discharge at a coastal site in the Gulf of Mexico. *J. Geophys. Res. Ocean.* **114**, (2009).
30. de Sieyes, N. R., Yamahara, K. M., Layton, B. a., Joyce, E. H. & Boehm, A. B. Submarine discharge of nutrient-enriched fresh groundwater at Stinson Beach, California is enhanced during neap tides. *Limnol. Oceanogr.* **53**, 1434–1445 (2008).
31. Spinelli, G. A. *et al.* Groundwater seepage into northern San Francisco Bay: Implications for dissolved metals budgets. *Water Resour. Res.* **38**, 12–19 (2002).
32. Strack, O. D. L. A single-potential solution for regional interface problems in coastal aquifers.

Water Resour. Res. **12**, 1165–1174 (1976).

33. Döll, P. & Fiedler, K. Global-scale modeling of groundwater recharge. *Hydrol. Earth Syst. Sci.* **12**, 863–885 (2008).
34. De Graaf, I. E. M., Sutanudjaja, E. H., Van Beek, L. P. H. & Bierkens, M. F. P. A high-resolution global-scale groundwater model. *Hydrol. Earth Syst. Sci.* **19**, 823–837 (2015).
35. Vries, J. J. de & Simmers, I. Groundwater recharge: an overview of processes and challenges. *Hydrogeol. J.* **V10**, 5–17 (2002).
36. Scanlon, B. R., Healy, R. W. & Cook, P. G. Choosing appropriate techniques for quantifying groundwater recharge. *Hydrogeol. J.* **10**, 18–39 (2002).
37. Sawyer, A. H., David, C. H. & Famiglietti, J. S. Continental patterns of submarine groundwater discharge reveal coastal vulnerabilities. *Science.* **10**, 705–708 (2016).
38. Younger, P. L. Submarine groundwater discharge. *Nature* vol. 382 121–122 (1996).
39. Burnett, W. C. *et al.* Quantifying submarine groundwater discharge in the coastal zone via multiple methods. *Sci. Total Environ.* **367**, 498–543 (2006).
40. Stewart, B. T., Santos, I. R., Tait, D. R., Macklin, P. A. & Maher, D. T. Submarine groundwater discharge and associated fluxes of alkalinity and dissolved carbon into Moreton Bay (Australia) estimated via radium isotopes. *Mar. Chem.* **174**, 1–12 (2015).
41. Taniguchi, M., Burnett, W. C., Cable, J. E. & Turner, J. V. Investigation of submarine groundwater discharge. *Hydrol. Process.* **16**, 2115–2129 (2002).
42. Hernández-Terrones, L. *et al.* Groundwater pollution in a karstic region (NE Yucatan): Baseline nutrient content and flux to coastal ecosystems. *Water. Air. Soil Pollut.* **218**, 517–528 (2011).
43. Lehner, B. & Grill, G. Global river hydrography and network routing: baseline data and new approaches to study the world's large river systems. *Hydrol. Process.* **27**, 2171–2186 (2013).
44. Hartmann, J. & Moosdorf, N. The new global lithological map database GLiM: A representation of rock properties at the Earth surface. *Geochemistry, Geophys. Geosystems* **13**, 1–37 (2012).
45. Moosdorf, N. & Oehler, T. Societal use of fresh submarine groundwater discharge: An overlooked water resource. *Earth-Science Rev.* **171**, 338–348 (2017).
46. Potter, P., Ramankutty, N., Bennett, E. M. & Donner, S. D. Characterizing the Spatial Patterns of Global Fertilizer Application and Manure Production. *Earth Interact.* **14**, 1–22 (2010).
47. Gleeson, T., Moosdorf, N., Hartmann, J. & van Beek, L. P. H. A glimpse beneath earth's surface: GLobal HYdrogeology MaPS (GLHYMPS) of permeability and porosity. *Geophys. Res. Lett.* **41**, 1–8 (2014).
48. Gleeson, T. *et al.* Mapping permeability over the surface of the Earth. *Geophys. Res. Lett.* **38**, 1–6 (2011).

49. Paytan, A. *et al.* Submarine groundwater discharge: An important source of new inorganic nitrogen to coral reef ecosystems. *Limnol. Oceanogr.* **51**, 343–348 (2006).

50. Maher, D. T., Santos, I. R., Golsby-Smith, L., Gleeson, J. & Eyre, B. D. Groundwater-derived dissolved inorganic and organic carbon exports from a mangrove tidal creek: The missing mangrove carbon sink? *Limnol. Oceanogr.* **58**, 475–488 (2013).

51. Krest, J. M., Moore, W. S., Gardner, L. R. & Morris, J. T. Marsh nutrient export supplied by groundwater discharge: Evidence from radium measurements. *Global Biogeochem. Cycles* **14**, 167–176 (2000).

52. Johannes, R. E. & Hearn, C. J. The effect of submarine groundwater discharge on nutrient and salinity regimes in a coastal lagoon off Perth, Western Australia. *Estuar. Coast. Shelf Sci.* **21**, 789–800 (1985).

53. Hu, C., Muller-Karger, F. E. & Swarzenski, P. W. Hurricanes, submarine groundwater discharge, and Florida's red tides. *Geophys. Res. Lett.* **33**, (2006).
