# Supplementary material for: Fresh groundwater discharge insignificant for the world’s oceans but important for coastal ecosystems
Source: Nat Commun. 2020 Mar 9;11:1260. doi: 10.1038/s41467-020-15064-8 (PMC7062736; doi:10.1038/s41467-020-15064-8)
Supplement: Supplementary file 3 — Description of Additional Supplementary Files [file 41467_2020_15064_MOESM3_ESM.pdf]

Description of Additional Supplementary Files

File name: Supplementary Data 1

Description: Results of model sensitivity analysis containing the modelled rates of coastal groundwater discharge for n=112 model runs with different values of model domain length, topographic gradient, groundwater recharge, permeability, aquifer thickness, permeability anisotropy, dispersivity and grid cell size.

File name: Supplementary Data 2

Description: Results of the model parameter space exploration containing the modelled rates of groundwater discharge for n=351 model runs with different values of topographic gradient, recharge volume and permeability.

File name: Supplementary Data 3

Description: The results of the geospatial analysis of controlling variables and the interpolated coastal groundwater discharge fluxes for the n=42086 global coastal watersheds. The data are supplied in shapefile format.
